# Rationally reduced libraries for combinatorial pathway optimization minimizing experimental effort

**DOI:** 10.1038/ncomms11163

**Published:** 2016-03-31

**Authors:** Markus Jeschek, Daniel Gerngross, Sven Panke

**Affiliations:** 1 Department of Biosystems Science and Engineering, ETH Zurich, Basel 4058, Switzerland

## Abstract

Rational flux design in metabolic engineering approaches remains difficult since important pathway information is frequently not available. Therefore empirical methods are applied that randomly change absolute and relative pathway enzyme levels and subsequently screen for variants with improved performance. However, screening is often limited on the analytical side, generating a strong incentive to construct small but smart libraries. Here we introduce RedLibs (Reduced Libraries), an algorithm that allows for the rational design of smart combinatorial libraries for pathway optimization thereby minimizing the use of experimental resources. We demonstrate the utility of RedLibs for the design of ribosome-binding site libraries by *in silico* and *in vivo* screening with fluorescent proteins and perform a simple two-step optimization of the product selectivity in the branched multistep pathway for violacein biosynthesis, indicating a general applicability for the algorithm and the proposed heuristics. We expect that RedLibs will substantially simplify the refactoring of synthetic metabolic pathways.

## Abstract (web summary)

Rational design in metabolic engineering is often difficult and limited to small screens, favouring construction of compressed smart libraries. Here the authors introduce RedLibs, an algorithm to design combinatorial RBS libraries to allow pathway optimization with minimal experimental resources.

## Graphical abstract

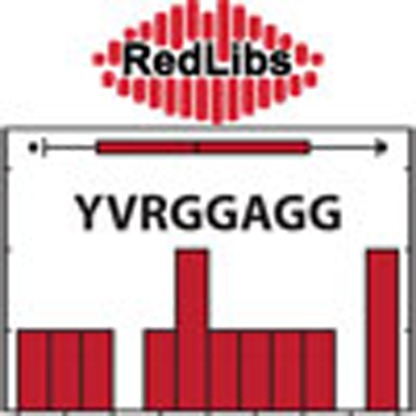

Synthetic biology is the forward engineering of biological systems, a process in which well characterized, predefined parts are combined in a rational manner to create a desired behaviour or function^1^. However, even well characterized parts can create unpredictable behaviour when introduced into new contexts, for instance when a recombinant pathway causes metabolic imbalances, thus leading to impaired growth and reduced product yields^2^. A possible reason for this dilemma are regulatory mechanisms that ensure optimal balance of a pathway in its natural context but are absent or non-functional in the new host^3^. In fact, recombinant pathways are frequently only poorly understood and important information is missing such as the specific activity of enzymes, intermediate toxicity and/or sinks and occurrence of branching points in the pathway, to name but a few. Consequently enzyme levels have to be optimized to improve the desired properties^4^.

This lack of knowledge prohibits strictly rational design methods, which are replaced by the creation of diversity and subsequent screening/selection, a process known as directed evolution^5^. This can lead to the emergence of significantly enhanced functions as demonstrated in a number of compelling cases^6^,^7^,^8^,^9^, and consequently be employed to identify optimal absolute and relative abundance of all enzymes constituting a pathway or the ‘metabolic sweet spot' within a complex expression level space of dimensionality *m* (number of proteins) and resolution *n* (expression levels tested per protein, Fig. 1). To alter and fine-tune expression levels several methods have been developed targetting gene dosage^10^,^11^, transcriptional regulation^12^,^13^,^14^, codon usage^15^,^16^ and protein half-life^17^. A promising alternative is the tailoring of translation rates by engineering the ribosomal-binding site (RBS)^18^,^19^,^20^. The latter has several compelling advantages: the number of base changes to access a large range of expression levels is small^20^, it allows independent adjustment for individual proteins in operons^18^ and RBS tailoring is applicable for a wide range of different hosts^21^. RBS engineering has been exploited by recruiting a set of experimentally pre-characterized RBSs for combinatory optimization of expression levels^18^. However, the effective RBS strength varies strongly for different genes due to differences predominantly in the 5′-coding region^20^, casting doubts on the general applicability of this strategy. Furthermore, each individual RBS combination needs separate cloning, which aggregates into a substantial effort when many components are optimized at high resolution. Both limitations can be overcome by a library design in which parts of the RBS are randomized. However, such an approach becomes very quickly limited by combinatorial explosion e.g., if a degenerate Shine–Dalgarno (SD) sequence of six or eight random bases (*N*s) is used: already a three-gene pathway leads to 6.9 × 10^10^ ((4^6^)^3^ for N_6_) or 2.8 × 10^14^ (N_8_) combinations, respectively. These numbers cannot be comprehensively evaluated experimentally and furthermore this pool would be highly redundant for weak RBSs whereas potentially more productive combinations including intermediate or strong RBSs would remain scarce.

Recently biophysical models were developed which calculate approximate rates for translation initiation (TIRs), the rate limiting step in translation^22^,^23^, strictly based on the RBS sequence facilitating the rational prediction of relative expression levels and the forward design of RBSs^20^,^24^,^25^,^26^ (see elsewhere for a review about available algorithms^27^). However, direct application of RBS prediction for refactoring of pathways is difficult since optimal expression levels are unknown and the predictive models are only approximate^20^ and do not include important factors such as gene dosage and promoter activity. This suggests that the exploration of a range of different TIRs rather than a specific value is the most advantageous strategy and combinatorial optimization to identify the metabolic sweet spot remains inevitable. Therefore smart libraries need to be generated that (i) cover a broad range of expression levels; (ii) are uniformly distributed; (iii) have a size that is amenable to screening; and (iv) are encoded by single degenerate sequences, which allow for experimental implementation by easy one-pot cloning procedures.

Finding globally optimal degenerate sequences is not a trivial problem since the number of degenerate sequences to be analysed scales even faster with the number of randomized bases than the aforementioned combinatorial explosion due to the 15-letter IUPAC base code of explicit and degenerate bases: e.g., for a degenerate RBS with eight Ns the number of degenerate sequences to be analysed for their TIR distributions amounts to 15^8^ (∼2.5 billion). Farasat *et al.*^21^ addressed this complexity using a genetic algorithm: starting from a predefined sequence the degenerate RBS is evolved by iterative diversity creation (mutation and recombination) and selection based on the TIR distribution of the resulting offspring sequences, typically over 50–100 cycles. This selection uses a set of predefined bins of exponentially increasing TIR strength and the respective distribution is evaluated based on the occupancy of these bins. This approach allows for optimization of RBSs in an evolutionary manner representing a good compromise between computational effort and generation of libraries that cover large ranges of TIRs, but does not deliver a global optimum and still retains a skew towards low-level TIRs due to the exponential binning.

Here we introduce the algorithm RedLibs (Reduced Libraries) that identifies globally optimal degenerate RBSs that uniformly span the entire TIR space in a linear manner. RedLibs compares the TIR distributions of all possible degenerate RBSs to a desired target distribution, leading to truly uniform predicted distributions that can be implemented by simple one-pot cloning protocols. We characterize these libraries by *in silico* and *in vivo* screening for two fluorescent proteins in different library sizes. Next, we demonstrate the applicability of RedLibs to a common metabolic engineering problem, the flux optimization around a branching point in a pathway, here for violacein biosynthesis. This example is representative for common low-throughput analysis methods and the pathway products are highly relevant because of their medical properties^28^,^29^,^30^. Finally, we exploit the high density of functional clones in RedLibs-derived libraries to develop a novel iterative method that allows for the identification a metabolic sweet spot with highly reduced analytical effort.

## Results

### Algorithm for rational reduction of library size

**Figure 2:**
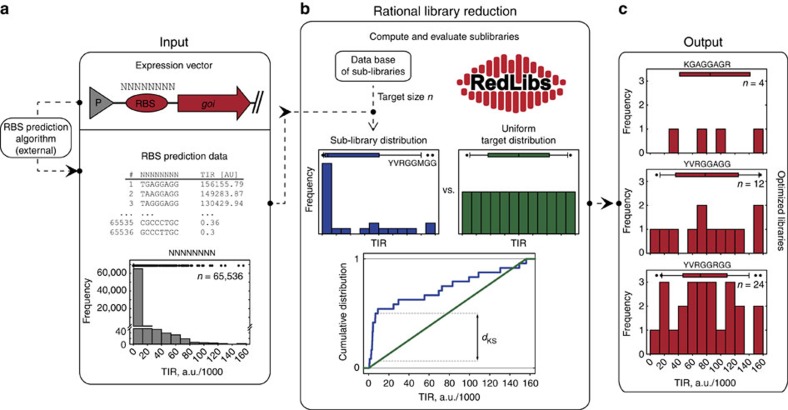
**Rational library reduction by RedLibs**. (**a**) An externally produced comprehensive input data set for a fully degenerate RBS library (consisting of *a* nucleotides, here *a*=8) for a specific gene of interest (*goi*, here exemplified with mCherry) contains *n*=4^*a*^ (65,536) sequence TIR (translation initiation rate) pairs with a strong skew to low TIR levels. (**b**) Based on this input data set RedLibs compares the TIR distributions of all possible sub-libraries of a user-defined size, which are organized in a database, with a desired target distribution (here: uniform) using the Kolmogorov–Smirnov distance *d*_KS_. (**c**) As an output the user receives a list of optimized sub-libraries (i.e., the 10 sub-libraries with the smallest *d*_KS_) along with the corresponding degenerate sequence for experimental implementation (shown here for mCherry and target sizes of 4, 12 and 24). The respective degenerate and explicit bases are given according to the 15-letter IUPAC nucleotide code. The boxplots over the histograms represent the median (box centre) and first/third quartile (box width) for the respective distribution with 10/90 percentiles displayed as whiskers and outliers as dots.

We developed RedLibs (freely available at: https://www.bsse.ethz.ch/bpl/software/redlibs), an algorithm that produces a partially degenerate RBS sequence coding for a library that allows for the most uniform sampling of the entire accessible translation level space for a target protein while concomitantly keeping the library at a small, user-specified size (Fig. 2). The algorithm requires a gene- and context-specific input data set of sequence TIR pairs generated by RBS prediction software like the RBS calculator^20^. For example, one can generate prediction data for a fully degenerate SD sequence (e.g., eight consecutive *N*s) in front of the gene for the red fluorescent protein mCherry and receive a list of 4^8^=65,536 RBS sequences with their corresponding predicted TIRs. As discussed, this initial RBS library is not well suited for implementation as it is too large and highly redundant for RBSs, which will lead to poor translation (e.g., for mCherry >99.5% of the library members exhibit a TIR of <10% of the entire range) (Fig. 2a). RedLibs uses this skewed input data set to produce smart sub-libraries of a user-specified target size that match the desired distribution (e.g., uniform distribution) as closely as possible and are encoded by a single degenerate sequence suitable for the cloning of a one-pot library.

These sub-libraries are generated by computing the TIR distributions of all possible partially degenerate sequences with a given target size (these sequences are obtained from a database which is part of RedLibs) and subsequently comparing them to the target distribution one by one. More precisely, this comparison is carried out by calculating the cumulative distribution function (CDF) for each sub-library and matching it with the CDF of the predefined target distribution (e.g., uniform over the entire TIR space). The largest deviation between both CDFs, the Kolmgorov–Smirnov distance *d*_KS_, represents a quantitative measure of resemblance between the distributions. Sequences with a low *d*_KS_ are more similar to the target distribution than those with a large *d*_KS_ (Fig. 2b). Finally, the distributions are ranked according to *d*_KS_ and the ranked list is returned to the user. These libraries represent globally optimal distributions for a given TIR prediction input data set and the desired library target size. This is exemplified for mCherry and the library sizes 4, 12 and 24 in Fig. 2c. Please note that in this case RedLibs had to evaluate 4.3, 25.7 and 70.2 million different possible sub-libraries, respectively, a task that cannot be coped with manually. Since RedLibs performs an exhaustive search which is in general a computationally intensive task we designed the underlying algorithm to be suitable for parallelization to minimize computing times and tested its performance for several tasks of different complexities (Supplementary Fig. 1).

The ease of DNA library generation is an important feature of the programme as it facilitates the one-pot generation of a combinatorial RBS library by simple PCR and/or assembly strategies. In summary, the smart composition of the RBS sub-library allows either to reduce the experimental effort while maintaining a high coverage of TIRs (and TIR combinations) or increasing the likelihood of finding meaningful TIR combinations with a given limited number of experiments that can be conducted due to throughput constraints of the experimental assay. The latter might also improve access to complicated pathway optimization approaches in which many pathway steps are balanced and/or high resolutions applied.

### Construction of reduced libraries for fluorescent proteins

**Figure 3:**
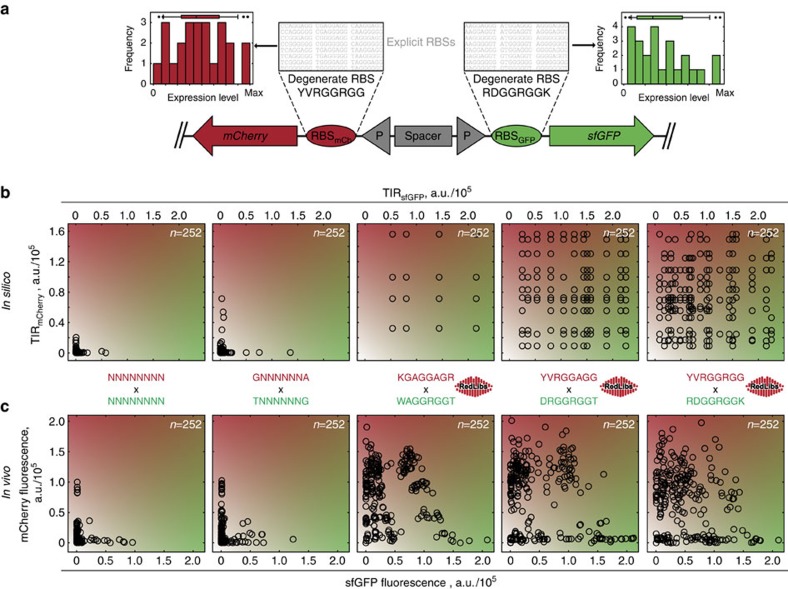
Performance of rationally reduced libraries for two fluorescent proteins. (**a**) A vector design (plasmid pMJ1) was chosen in which the genes for sfGFP and mCherry are placed under control of opposing constitutive promoters separated by a spacer to allow independent randomization of both RBSs. (**b**) The performance of irrationally randomized RBSs (*N*_6_ × *N*_6_ and *N*_8_ × *N*_8_) as well as of three libraries optimized by RedLibs (sizes 4 × 4, 12 × 12 and 24 × 24) were compared by random *in silico* picking (252 picks). (**c**) The behaviour of the rationally reduced libraries was analysed *in vivo* by measuring the cell-specific green and red fluorescence of 252 independent cultures of *E. coli* for each library (*N*_6_, *N*_8_, 4, 12 and 24).

To evaluate the quality of the library distributions produced by our algorithm we conducted a proof of principle experiment in which we concomitantly randomized the RBSs of two fluorescent proteins, superfolder green fluorescent protein (sfGFP) and the red fluorescent protein mCherry (Fig. 3). For this, we constructed plasmid pMJ1 containing the two corresponding genes placed under the control of two copies of the same constitutive promoter in opposite directions separated by a piece of insulating silent DNA to prohibit undesired transcriptional interaction. In front of each gene (7 bp upstream of the start codon) the degenerate RBSs were inserted (Fig. 3a). We applied two different RBS diversification strategies: an unstructured randomization in which six or eight nucleotides were fully randomized (*N*_6_ or *N*_8_) as well as diversification using smart RBS libraries with target sizes of 4, 12 and 24 members produced by RedLibs to resemble a uniform distribution. We initially performed an *in silico* screening approach to mimic an actual screen with low throughput by randomly picking 252 TIR pairs for the two fluorescent proteins from the respective libraries (Fig. 3b). As expected for low numbers of picks the fully degenerate libraries deliver only combinations of low TIR levels for both proteins, representing unsatisfactory coverage of the translation level space whose boundaries are defined by the minimum and maximum predicted TIR for each gene. By contrast, the libraries produced by RedLibs achieve good coverage of the entire space already at a small library size (4 × 4) and very good coverages for the 12 × 12 and 24 × 24 libraries indicating a highly improved representation of intermediate and high TIR levels and their combinations. Please note that whereas for the reduced libraries only 48 (4 × 4 library), 432 (12 × 12 library) and 1,726 (24 × 24 library) picks would be necessary to achieve a statistical likelihood of >95% of covering all possible combinations, the fully degenerate libraries would require approximately 5.0 × 10^7^ (*N*_6_ × *N*_6_ library) and 1.3 × 10^10^ (*N*_8_ × *N*_8_ library) picks to reach the same coverage^31^. Based on these *in silico* results we implemented the same system *in vivo* and measured the cell-specific green and red fluorescence of 252 clones (three microtiter plates) for each library (Fig. 3c). The experimental results showed a great qualitative resemblance between the *in silico* predicted and the *in vivo* observed behaviour for all libraries: whereas candidates picked from the fully degenerate RBS libraries failed to span the whole two-dimensional expression space, the reduced libraries showed a much better coverage for nearly all expression level combinations. Only for combinations of very high expression levels for both proteins no clones were found in the anticipated region (upper right corner of the graphs, Fig. 3c). Deep-sequencing analysis confirmed the presence of sequences that were predicted to lead to combinations of two high TIR values within the libraries which suggested that very high synthesis levels of both proteins are incompatible with the synthesis capacity of the cell. This was confirmed by analysis of a manually cloned mutant containing the highest TIR combination for the sfGFP and mCherry RBS, which showed a strong downshift in the sfGFP signal as compared with the clone containing the identical RBS for sfGFP but a weak RBS for mCherry (Supplementary Fig. 2) pointing to mutual influences when combining multiple strong RBSs.

### Optimization of violacein production using smart libraries

**Figure 4:**
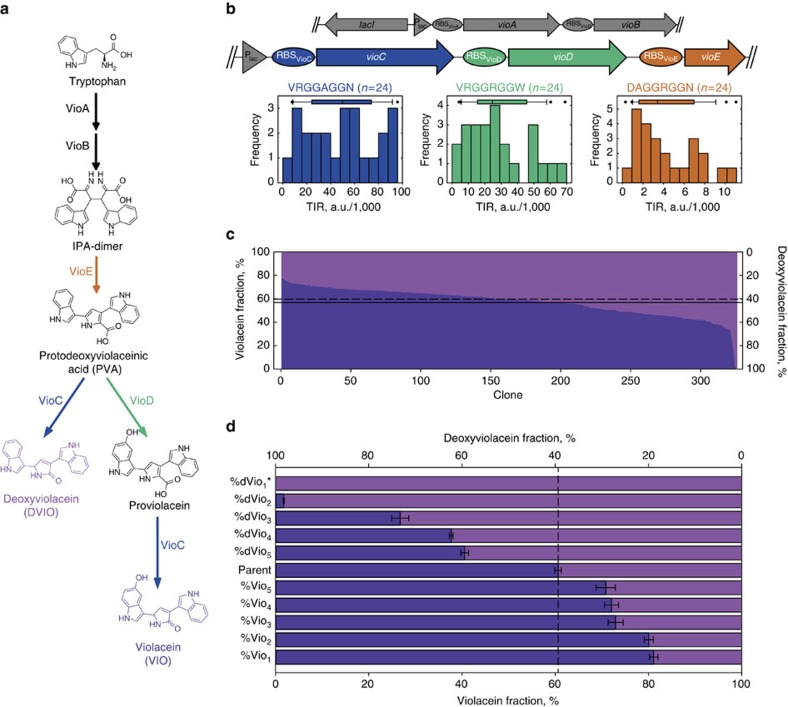
**Rationally reduced library for optimization of violacein production in** ***E. coli***. (**a**) The pigment violacein (VIO) is produced in five heterologous reactions (catalysed by VioABEDC) from L-tryptophan. The branched nature of the pathway leads to the formation of other pigments including the main byproduct deoxyviolacein (DVIO). (**b**) For pathway optimization the genes *vioC*, *vioD* and *vioE* were organized in an operon-like structure (plasmid pMJ3). The expression levels were uniformly randomized by implementing a rationally reduced library with a target size of 24 produced by RedLibs out of an initial *N*_8_ data set for each of the respective proteins. VioA and B were synthesized from a helper plasmid (pMJ2). (**c**) The rationally reduced RBS library allowed for the creation of a phenotypic variety of clones with distinctly altered product spectra as compared to the library average (solid line) and to the parent clone (pMJ3 with unaltered RBSs, dashed line). With a limited screening effort (372 clones in 96-well microtiter plates) mutants with significantly altered selectivity for the production of VIO and DVIO could be obtained. Please note that of the 372 clones analysed 47 were excluded from the figure because they did not produce detectable amounts of either pigment. (**d**) Quantitative pigment extraction of the five clones with the highest selectivity for either VIO (%Vio_1–5_) or DVIO (%dVio_1–5_). Data represents the mean of four independent replicate cultures with standard deviation. *Please note that clone %dVio_1_ had an extra stop codon inserted in *vioD* and can therefore be considered a knockout mutant for this gene leading to pure DVIO production (compare **a**).

To demonstrate the utility of RedLibs for pathway optimization we chose the five-step synthesis pathway for the pigment violacein from L-tryptophan catalysed by VioABEDC. This pathway has been characterized *in vitro* as well as *in vivo* in different organisms^11^,^32^,^33^,^34^,^35^,^36^,^37^,^38^. Due to its branched nature after the intermediate protodeoxyviolaceinic acid and the occurrence of at least two products (violacein and deoxyviolacein) which both require the activity of the same enzyme (VioC) after the branching point (Fig. 4a), we considered it a good test bed for expression level optimization, as the production of violacein with a minimum of deoxyviolacein formation requires on the one hand much VioC to prevent accumulation of proviolacein and to guarantee a certain productivity, but on the other hand little VioC to prevent precursor drainage towards deoxyviolacein. Clearly, a fine-tuned orchestration of VioC and VioD needs to be established to improve the purity of violacein without sacrificing too big a portion of its absolute production level. Furthermore, VioE is a crucial player in determining product spectrum and amount since it directly affects the abundance of protodeoxyviolaceinic acid.

We therefore reasoned that a rationally reduced RBS library would allow tuning the fluxes around the branching point and consequently isolation of clones with a drastically altered product spectrum at a minimal screening effort by randomizing the expression level of the final three enzymes (VioCDE) of the violacein biosynthesis pathway using plasmid pMJ3. Supply of protodeoxyviolaceinic acid as the substrate of the branching point was ensured by fixing the expression of the first two genes (*vioAB*) from a helper plasmid (pMJ2) with higher copy number (pBR322 ori) when compared to the designated library plasmid for expressing *vioCDE* (p15A ori) (Fig. 4b). This setup was primarily designed to influence the ratio between the two main products violacein and deoxyviolacein (selectivity) rather than maximizing the overall yield for one or both of the pigments (quantity) with the main goal of producing high purities of violacein, as pure deoxyviolacein could be obtained from a simple *vioD* knockout strain.

Out of the three initial prediction data sets (one for the RBS of each gene) derived from a full randomization of the SD sequence (*N*_8_), RedLibs produced three rationally reduced libraries (target size: 24, maximum number of different combinations in the library: 24^3^=13,824) which exhibited a good uniform distribution of predicted TIRs over the whole range of possible TIR levels (Fig. 4b). These libraries were inserted in front of the three genes by extension PCR and the resulting pathway library with concomitantly varied levels for VioC, VioD and VioE was expressed in *Escherichia coli*. A subset of 372 clones (∼2.7%) of this library was grown in microtiter plates and the produced pigments (violacein and deoxyviolacein) were semi-quantitatively extracted and quantified by HPLC (Fig. 4c). This first and rough characterization was applied to obtain an overview of pigment distributions and identify promising combinations for further analysis. Although the exact values for pigment production cannot be faithfully obtained from the semi-quantitative process, the results tentatively suggested that it was possible to tune the ratio of the two pigments quite finely despite the small number of tested clones, as the library exhibited broad variability in several aspects. Of the 372 analysed clones, 325 producers showed a strong diversity in absolute pigment amounts produced. Despite the comparably low throughput of the applied screening assay it was possible to identify clones producing a 240%, 420% and 190% increased pigment level relative to the library average for violacein, deoxyviolacein and total pigment (i.e., violacein + deoxyviolacein), respectively (Supplementary Fig. 3a). Moreover, a wide variability of product selectivity was observed, with violacein making up between 0 and 77% and deoxyviolacein making up between 23 and 99% of total pigments (Fig. 4c).

A fraction of 47 of the 372 clones did not produce any detectable amounts of violacein or deoxyviolacein. Possible reasons for this lack of productivity include primarily undesired mutations in the coding region of the violacein genes, but also the occurrence of unproductive combinations of RBSs for the genes of the final steps in the pathway.

Subsequently we chose the five best clones with regard to each of the following objectives: high absolute violacein level (Vio_1–5_), high absolute deoxyviolacein level (dVio_1–5_), high total pigment level (TP_1–5_), high selectivity for violacein (%Vio_1–5_) and high selectivity for deoxyviolacein (%dVio_1–5_). These 25 clones were re-grown in four independent cultures each and afterwards analysed by quantitative pigment extraction and HPLC. All clones showed superior production behaviour as compared with the parent plasmid (pMJ3 equipped with the initial RBS in front of the three genes as in Addgene plasmid #40782 (ref. 39)). The best clones chosen for improvement in absolute pigment production showed an increase by 34% for violacein, 260% for deoxyviolacein and 44% for total pigment over the parent strain, respectively (Supplementary Fig. 3b). More importantly, the mutants selected for higher selectivity for one pigment showed an increase in violacein fraction from 61 to 81% and in deoxyviolacein fraction from 39 to >99% (i.e., no detectable contamination with violacein) (Fig. 4d). It should be mentioned that, as expected (see above) the clone with the highest purity for deoxyviolacein (%dVio_1_) was found to contain an in-frame stop codon mutation after 157 residues in the *vioD* gene and can be therefore considered a knockout for the respective enzyme leading to exclusive production of deoxyviolacein.

### Analysis of predicted TIRs and improved library design

**Figure 5:**
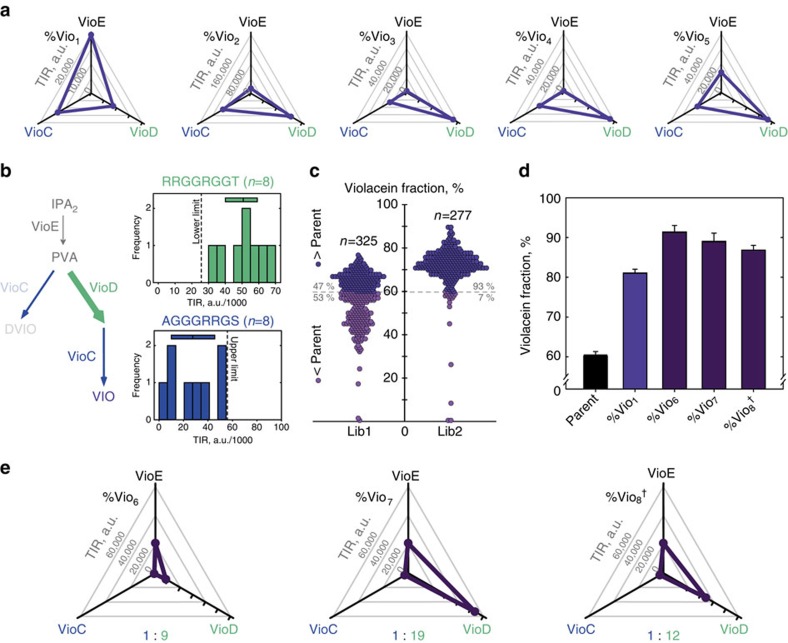
Improved library design for high violacein selectivity. (**a**) The TIRs for the clones with the highest violacein selectivity (clones %Vio_1–5_; prediction value according to ref. 26) from the initial library suggest a crucial role of the ratio of TIR_VioC_ and TIR_VioD_ in the lower stoichiometric range for high violacein selectivity, with a tendency towards a higher value for TIR_VioD_ than for TIR_VioC_. (**b**) Graphical representation of the rationale behind the second library design by RedLibs. TIRs for VioD are uniformly distributed in the upper range of the accessible TIR space whereas for VioC the lower range is uniformly covered. (**c**) The resulting library was nearly exhaustively screened revealing a strong tendency to high violacein selectivity relative to the first library as well as the parent mutant (plasmid pMJ3 with unaltered RBSs, dashed line). (**d**) From the improved library the clones with the highest violacein purity were obtained (%Vio_6–8_) and analysed in detail by quantitative pigment extraction. Bars represent the average violacein fraction of four independent replicate cultures with standard deviation. (**e**) The RBSs of %Vio_6-8_ were determined and the corresponding TIRs calculated. ^†^Please note that clone %Vio_8_ was independently found three times in the improved library.

After the identification of clones with improved violacein selectivity from the first library we determined the sequence of the corresponding RBSs for *vioC*, *vioD* and v*ioE* and obtained TIR predictions for each clone by taking the operon context into account^20^,^26^. In Fig. 5a the predicted TIR values for the five clones with the highest selectivity for violacein (%Vio_1–5_) are presented. As mentioned previously this group represents the most challenging objective since a tight balance of pathway enzymes is necessary to obtain high selectivity for violacein. Interestingly, the ratios between the TIRs for VioC and VioD seemed to be exclusively in the lower range within this group and clearly pointed to an excess of VioD over VioC in four of the five cases. By contrast, the TIR for VioE did not seem to be a crucial determinant for high violacein selectivity since it varied significantly among the five analysed clones, both absolute and in relation to the other two TIRs. For the clones with the highest selectivity for deoxyviolacein overall an inverted trend (i.e., TIR_VioC_ > TIR_VioD_) was observable (Supplementary Fig. 4).

Given this observed pattern, there were two options to proceed towards increasing the selectivity further: either to continue screening the original library or the construction of a small new library whose target distribution of TIRs was tailored towards the observed pattern using the RedLibs algorithm. Since exhaustive screening of this first library would have required considerable effort, we decided instead to pursue the latter option and designed a second, improved library to produce clones with low ratios between TIR_VioC_ and TIR_VioD_ with a tendency for VioD > VioC. The resulting library should reflect the trends observed in the small initial screening campaign and lead to the identification of superior mutants with a significantly increased frequency, again within a limited screening effort. Therefore the algorithm was set to a uniform target distribution within the lower part of the entire TIR range for VioC and the upper part of TIRs for VioD (Fig. 5b). The target size for the library was set to eight resulting in 8 × 8=64 potential combinations of expression levels for VioC and VioD. For VioE, we retained the RBS that had been found in the clone with the highest absolute concentration of violacein (Vio_1_), thus ensuring a high flux to the branching point. The resulting fine-tuned library could be nearly exhaustively screened (analysis of 279 clones (three 96-well plates), statistical coverage of >98.7% of all potential RBS combinations in the respective library^31^), and in fact the selectivity for violacein increased further: >93% of library members demonstrated a higher selectivity for violacein than the parent clone (with pMJ3) before RBS engineering indicating that the design principles deduced from the first library were valid (Fig. 5c). Subsequent analysis of the five best clones from the fine-tuned library (replicate cultivation and quantitative extraction) revealed significantly improved selectivity (clones %Vio_6–8_, Fig. 5d) as compared with the producer with the highest selectivity isolated from the first library (%Vio_1_): 91% versus 81%. Sequencing revealed that three of the clones had in fact an identical RBS combination and were therefore combined to the clone %Vio_8_ in Fig. 5. To exclude that this repeated finding is due to a bias in the library we sequenced both a pool of the initial library as well as other individual clones which confirmed that we had isolated the same combination repeatedly because of the high selectivity value. This is not surprising given that our screening included oversampling by a factor greater than 4.3. Evaluating the predicted RBS strength for %Vio_6–8_ in the operon context^21^ again confirmed that the clones suited with the library design principle of low ratios of TIR_VioC_ over TIR_VioD_, with the most selective clone showing a 1:9 ratio of predicted TIRs (Fig. 5e).

Consequently, RedLibs allowed for the design of a smart two-step optimization scheme which led to efficient improvement of the targeted pathway and can be applied to the iterative synthetic biology design-build-test cycle.

## Discussion

Relative and absolute balancing of expression levels within an engineered pathway of interest in a strictly experimental manner remains a major challenge in metabolic engineering. Smartly designed combinatorial libraries, for instance of RBSs, can increase the number of meaningful library variants and are thus a highly useful tool to improve the outcome of the inevitable directed evolution approaches. Here, we develop, test and apply the algorithm RedLibs which allows efficient exploitation of a biophysical model^20^ to improve RBS library design. By comparison of cumulative distribution functions RedLibs produces rationally reduced sub-libraries, that (i) are small enough to meet the throughput of the respective screening, (ii) uniformly span the entire range of accessible expression levels (or TIRs) and (iii) are encoded by single degenerate sequences that allow implementation in simple a one-pot cloning procedure. Moreover, in contrast to alternative approaches which use a genetic algorithm to randomly create diversity^21^ RedLibs is capable of identifying the globally optimal libraries within the given data set and will reproducibly deliver identical results for a given task.

Due to the high proportion of meaningful sequences within the libraries produced by RedLibs we were able to demonstrate that it is possible to extract patterns from promising candidate clones in a first step (initial library) and subsequently feed this information back into the algorithm which in turn allowed us to design a second, improved library for a given task to obtain further improved clones at very limited combined screening effort. These iterative heuristics are an important element of the presented work since they allow keeping the overall experimental effort low while still leading to excellent solutions for the ‘sweet spot' problem.

The identified changes that led to the improved behaviour in the case of violacein selectivity may seem somewhat expected, especially since the tackled pathway is well characterized and the sequential order and topology of the enzymes is known. However, the exact measures that have to be taken to actually guide the flux around the branching point into the desired direction are non-trivial since important parameters like promoter strength, copy number effects, differences in specific activity and *K*_m_, potential regulation, unknown side-reactions, and so on are unknown. Moreover, RedLibs and the corresponding heuristics that we proposed in this work can likewise be applied to uncharacterized pathway operons. In this case a smart screening strategy might even lead to a better understanding of the respective pathway potentially in combination with metabolic flux models.

Finally, we would like to point out that RedLibs is not restricted to uniform target distributions and can easily be adjusted to produce for instance a Gaussian distribution around a user-specified target value. This could for example be useful for fine-tuning purposes in cases where an approximate optimum is known. Moreover, the algorithm is not restricted to RBS prediction sets as input data but can virtually use any sequence-value pair to optimize distributions be it predicted or experimentally determined data.

We anticipate that RedLibs will be a helpful contribution to the aforementioned fields of application in comparison to random approaches as well as to existing methods.

## Methods

### Computational implementation of RedLibs

RedLibs was developed in the integrated development environment (IDE) XCode (version 4.6) using C*++* as programming language. Parallelization of the algorithm was implemented using OpenMPI (version 1.4.3). RedLibs uses a database of sequence combinations which were generated using the programming language R (version 2.14.1) in the IDE RStudio (version 0.98.501). For the detailed script please refer to Supplementary Software or https://www.bsse.ethz.ch/bpl/software/redlibs. Additionally, the source code was deposited under: https://github.com/dgerngross/RedLibs. The input data for RedLibs was externally generated using the RBS Library Calculator^20^ (https://salislab.net/software/) in the ‘Predict: RBS Library' mode using either six or eight consecutive *N* bases generating 4,096 or 65,536 sequence TIR pairs. The corresponding RBS sequences including relevant up- and downstream regions for all randomized genes in this work are provided in Supplementary Table 1.

### Evaluation of library distributions

RedLibs uses the CDF of each evaluated library and compares it with the cumulative distribution function of the desired target distribution. As a criterion of evaluation the Kolmogorov–Smirnov distance *d*_KS_ is used which is defined as the maximum absolute difference between the actual cumulative distribution function *F*_actual_(*x*) and the target distribution function *F*_target_(*x*)^40^,^41^:


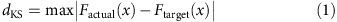


where *F*(*x*) is derived from a distribution of *n* numerical values according to the following equation in which *H*_*n*_ denotes the number of values smaller than *x* (ref. 42):


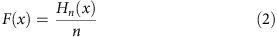


Consequently distributions with a high degree of resemblance to the target distribution will exhibit a low *d*_KS_ value and vice versa. For an exemplary illustrative description of the evaluation process please refer to Supplementary Fig. 5.

### Strains and growth media

*E. coli* TOP10 (F^−^ *mcrA Δ(mrr-hsdRMS-mcrBC) ϕ80lacZΔM15 ΔlacX74 nupG recA1 araD139 Δ(ara-leu)7697 galE15 galK16 rpsL*(Str^R^) *endA1 λ*^−^, Thermo Fisher Scientific) was used for cloning, expression of fluorescent proteins and for violacein biosynthesis. For the isolation of plasmids with methylation sensitive restriction sites *E. coli* dam^−^/dcm^−^ (*ara*^−^*14 leuB6 fhuA31 lacY1 tsx78 glnV44 galK2 galT22 mcrA dcm*^−^*6 hisG4 rfbD1* R(*zgb210::Tn10*) TetS *endA1 rspL136* (Str^R^) *dam13::Tn9* (Cm^R^) *xylA*^−^*5 mtl*^−^*1 thi*^−^*1 mcrB1 hsdR2,* New England Biolabs) was used.

For expression of the fluorescent proteins sfGFP and mCherry cells were grown in M9 mineral medium^43^ supplemented with 10 g l^−1^ glucose, 0.01% thiamine, 1 ml l^−1^ trace element solution^44^, 0.1 g l^−1^ L-leucine, 0.03 g l^−1^ L-isoleucine and 0.15 g l^−1^ L-valine, 50 mg l^−1^ kanamycin and 25 mg l^−1^ streptomycin. For the production of violacein pre-cultures were grown in Luria–Bertani (LB) medium^43^ supplemented with 5 g l^−1^ glucose, 50 mg l^−1^ kanamycin and 50 mg l^−1^ carbenicillin. Main cultures were grown in a modified ZYM-5052 medium^45^ lacking lactose as autoinduction agent but containing 50 mg l^−1^ kanamycin and 50 mg l^−1^ carbenicillin. Induction was performed by the addition of isopropyl-β-D-thiogalactopyranosid (IPTG) to a final concentration of 500 μM.

### Vectors for fluorescent proteins and library construction

Vector pMJ1 for fluorescent protein synthesis was constructed according to the Standard European Vector Architecture (SEVA, ref. 46) format and contained a kanamycin resistance cassette, a p15A origin of replication and the default SEVA multiple cloning site. The expression cassette containing the genes for sfGFP and mCherry was obtained by commercial gene synthesis (Life Technologies, Regensburg, Germany, Supplementary Table 2) and cloned into the vector backbone using the *Hind*III and *Eco*RI sites, resulting in pMJ1. It contained the two genes facing outward in opposing directions from a regulatory region located between them. Each gene was under the control of a separate copy of the constitutive promoter BBa_J23118 (BioBricks parts registry). To ensure independent transcription the two promoters were separated by a 70 bp unstructured stretch of silent spacer DNA (BBa_B0040). To demonstrate the general applicability of our approach irrespective of the initial 5′-UTR of the respective gene we equipped the gene for sfGFP with the −35 bp region upstream of the start codon of the β-lactamase gene from pUC18 (ref. 47) and the gene for mCherry with the corresponding region from commercially available pET vectors^48^. Plasmids used in this study are listed in Supplementary Table 3.

RBS libraries were constructed by PCR extension^49^ of overlapping degenerate oligo pairs 1+2 (leading to an RBS library of *N*_8_ × *N*_8_), 3+4 (*N*_6_ × *N*_6_), 5+6 (4 × 4 combinations), 7+8 (12 × 12 combinations) and 9+10 (24 × 24 combinations), and subsequently digesting (with *Xba*I and *Sph*I) and ligating the resulting DNA fragment into the parent vector treated with the same restriction enzymes. A graphic depiction of the XFP library cloning procedure including important sequences is provided in Supplementary Fig. 6 and the oligonucleotides used are listed in Supplementary Table 4.

### *In silico* screening

The *in silico* picking experiment to retrieve hypothetical clones from the fluorescent protein libraries was performed using the programming language *R* (version 2.14.1) in the IDE RStudio (version 0.98.501). Pairwise combinations of predicted TIRs for sfGFP and mCherry were randomly picked from the different libraries at identical probabilities for each TIR value contained in the respective library (e.g., for the library with the size of 4 a 25% probability was applied for each of the four TIRs). For comparability the same number of randomly selected TIR pairs (252 in all experiments) per library were produced as clones analysed in the *in vivo* experiment. For random picking the ‘sample()'-function, a standard function in R, was used (Supplementary Methods).

### Fluorescence measurements

For the quantification of fluorescent proteins 96-deepwell plates containing 1 ml M9 mineral medium per well were inoculated from single colonies and incubated at 37 °C and 300 r.p.m. (5 cm shaking diameter) overnight. Afterwards these pre-cultures were used for the inoculation (1/100 dilution) of a working plate containing fresh medium and main cultures were incubated for seven hours (37 °C, 300 r.p.m.). Cells were harvested by centrifugation (10 min, 3,220*g*), resuspended in 1 ml of phosphate buffered saline (PBS), and incubated at 4 °C overnight on a table top shaker to ensure full maturation of fluorescent proteins. Subsequently 200 μl of the cell suspensions were transferred into a 96-well flat bottom plate and the optical density at 600 nm (OD_600_) as well as the fluorescence quantification were performed in a Tecan Infinite M200 plate reader. The quantification of sfGFP and mCherry was carried out at *λ*_Ex_=488 nm/*λ*_Em_=530 nm and *λ*_Ex_=579 nm/*λ*_Em_=616 nm, respectively. The fluorescence values were corrected for a well containing PBS only (blank) and normalized for the OD_600_ to retrieve cell-specific values. To ensure plate-to-plate comparability reference clones were included in three replicates each for low fluorescence in both channels (control plasmid lacking the genes for sfGFP and mCherry) as well as for high green and high red fluorescence (strong RBS for sfGFP or mCherry, respectively).

### Deep sequencing of fluorescent protein libraries

To confirm the presence of combinations of RBSs with high TIR values we performed deep sequencing of RBS libraries. Specifically, aliquots of cultures of the 252 tested clones were pooled, plasmid DNA was extracted, and the RBS regions were PCR amplified using oligo pair 11+12. The PCR product was gel purified and then submitted to next generation sequencing on an Illumina MiSeq platform (Illumina RTA Version: 1.18.54, Sequencer: GFB MiSeq (Yoda), Run type: PE-250). Results were analysed by an in-house developed software for NGS data analysis.

### Cloning of violacein operon and library generation

Plasmids for violacein biosynthesis were constructed according to the SEVA^46^ format. The helper plasmid pMJ2 for the expression of *vioAB* contained a β*-*lactamase gene, a pBR322 origin of replication and the default SEVA multiple cloning site.

The *vioAB* cassette was PCR amplified from BBa_J72214-BBa_J72090 (Addgene plasmid #40782, ref. 39) using primers 13 and 14 and fused to the lactose-inducible promoter P_*lac*_ by overlap extension PCR^49^ which was amplified using primers 15 and 16 and plasmid pCK01 (ref. 50) as template. The fusion product was cloned into the SEVA backbone by restriction digest (*Hind*III and *Bam*HI) and ligation. To avoid a high degree of leaky expression from P_*lac*_ due to an unfavourable ratio of promoter copies to transcriptional repressor a copy of the *lacI* gene was amplified from pET30b(+) (Novagen) with primers 17 and 18 and was then introduced upstream of P_*lac*_*-vioAB* pointing into the opposite direction by restriction digest (*Spe*I and *Hin*dIII) and ligation.

The designated library plasmid pMJ3 for the expression of *vioCDE* contained a kanamycin resistance cassette, a p15A origin of replication, and the default SEVA multiple cloning site under the control of P_*lac*_ from pCK01. The *vioCDE* genes were obtained by commercial gene synthesis (Life Technologies, Regensburg, Germany) and inserted into the SEVA backbone by digest (*Bgl*II and *Kpn*I) and ligation. pMJ3 was equipped with the RBSs in front of the three genes as in Addgene plasmid #40782 (ref. 39). The first RBS library was produced by generation of three PCR products using primers 19 and 20, 21 and 22, and 23 and 24, respectively, and subsequent overlap extension PCR to fuse the *vioCDE* cassette with degenerate RBSs for all three genes together to one linear fragment that was ultimately cloned into the parent vector (*Bgl*II and *Kpn*I). Likewise, the second library randomizing only the RBSs for *vioC* and *vioD* was cloned using the primer pairs 25 and 26, 21 and 22, and 27 and 24 to generate PCR fragments before fusion and cloning. A graphic depiction of the cloning procedure including important sequences is provided in Supplementary Fig. 7.

### Expression of violacein genes and pigment production

For expression of the violacein biosynthesis genes 96-deepwell plates containing 500 μl of LB per well were inoculated from single colonies and incubated at 37 °C and 300 r.p.m. overnight. An aliquot of 30 μl of these pre-cultures was then used to inoculate a main culture plate containing 1 ml of modified ZYM-5052 medium per well. After 210 min of cultivation (37 °C, 300 r.p.m.) the violacein biosynthesis genes were induced by addition of IPTG to a final concentration of 500 μM and expression was carried out for another four hours (37 °C, 300 r.p.m.) before harvest.

### Violacein extraction and quantification

Isolation of pigments was performed by ethanol extraction according to a modified version of an earlier protocol^32^. For semi-quantitative extractions meant to allow a rough classification of extracts, 500 μl of culture were directly added to 1 ml of pure ethanol and pigments were extracted from the cells for 20 min by vigorous agitation (1,750 strokes min^−1^) in a homogenizer (2010 Geno/Grinder). After centrifugation (21,130*g*, 5 min) the supernatant with the pigments was stored at −20 °C until quantification. Quantitative extractions were performed in two cycles to ensure full pigment transfer into the extract and accurate quantification: cells from 1 ml of culture were harvested by centrifugation (6,000*g*, 2 min) and resuspended in 1 ml of pure ethanol. Extraction was performed as before (1,750 strokes min^−1^, 20 min), the supernatant was collected after centrifugation and the pellet was applied to a second extraction step with 1 ml of fresh ethanol. The two supernatant fractions were pooled and stored at −20 °C until quantification of pigments by HPLC.

Chromatographic separation of violacein and deoxyviolacein was achieved using a ReproSil-Pur C18-AQ column (5 μm, 250 × 4 mm; Dr Maisch GmbH, Germany) on an Agilent 1200 Series HPLC machine with 50% ethanol as a mobile phase applying a flow rate of 1 ml min^−1^ at 30 °C. Violacein and deoxyviolacein were available as pure standards (BioAustralis Fine Chemicals, Australia) for reference and quantification was carried out by measuring the absorbance in the diode array detector at 575±8 nm (ref. 35).

## Additional information

**Accession codes:** Nucleotide sequences from deep sequencing were deposited in the National Center for Biotechnology Information Sequence Read Archive under accession number SRX1584490.

**How to cite this article:** Jeschek, M. *et al.* Rationally reduced libraries for combinatorial pathway optimization minimizing experimental effort. *Nat. Commun.* 7:11163 doi: 10.1038/ncomms11163 (2016).

## Supplementary Material

Supplementary Information Supplementary Figures 1-7, Supplementary Tables 1-4, Supplementary Methods and Supplementary References

Supplementary Software Sequence-numerical value pair lists of fully degenerate sequences are used as input to calculate smart libraries with a desired distribution encoded by a reduced degenerate sequence. Source code files (C++) for the RedLibs algorithm v1.0.0 including file description are provided. A frequently updated version can be found under the link provided in the Methods section

## Figures and Tables

**Figure 1:**
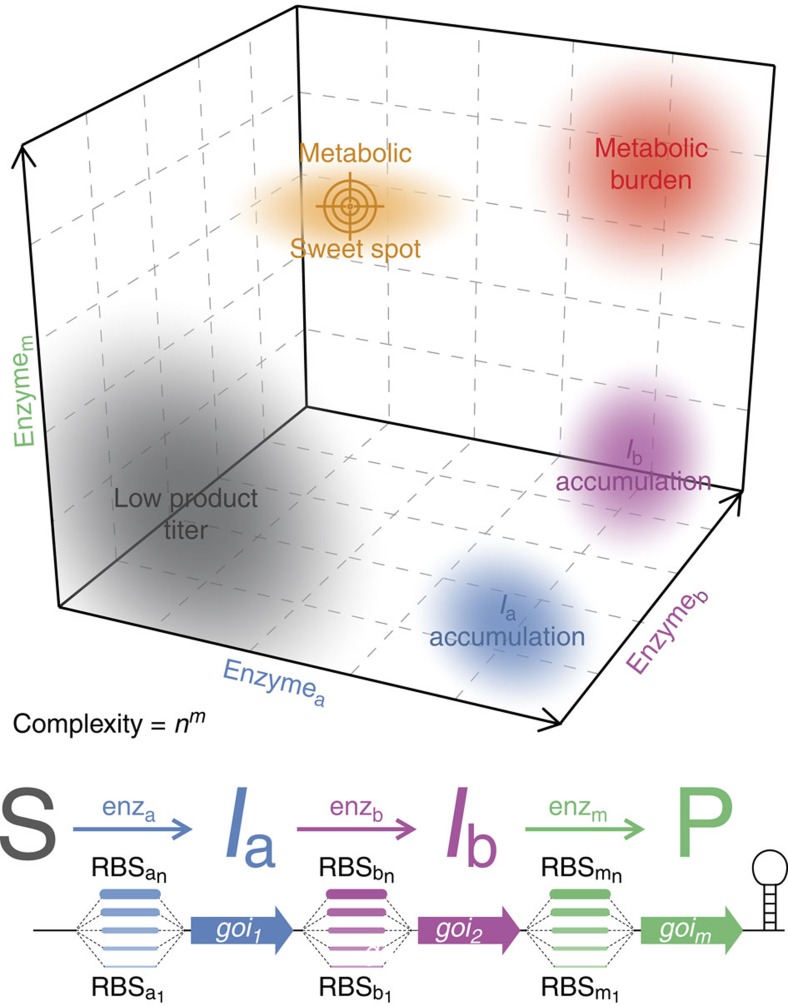
Multidimensional expression level optimization. To optimize the flux within a pathway composed of *m* enzymatic steps the expression level of each component has to be randomized concomitantly (e.g., by RBS engineering) to identify the metabolic sweet spot in a space that is otherwise dominated by regions of undesired behaviour including low product titers, accumulation of intermediates (*I*_a_, *I*_b_) or metabolic burden. The number of different expression levels *n* per pathway component determines the resolution of the search leading to a problem of quickly rising complexity for higher numbers of pathway components *m* and higher resolution *n*. The complexity of the search (i.e., the number of possible expression level combinations) can be calculated according to *n*^*m*^.
