# Supplementary material for: Rationally reduced libraries for combinatorial pathway optimization minimizing experimental effort
Source: Nat Commun. 2016 Mar 31;7:11163. doi: 10.1038/ncomms11163 (PMC4821882; doi:10.1038/ncomms11163)
Supplement: Supplementary Information — Supplementary Figures 1-7, Supplementary Tables 1-4, Supplementary Methods and Supplementary References [file ncomms11163-s1.pdf]

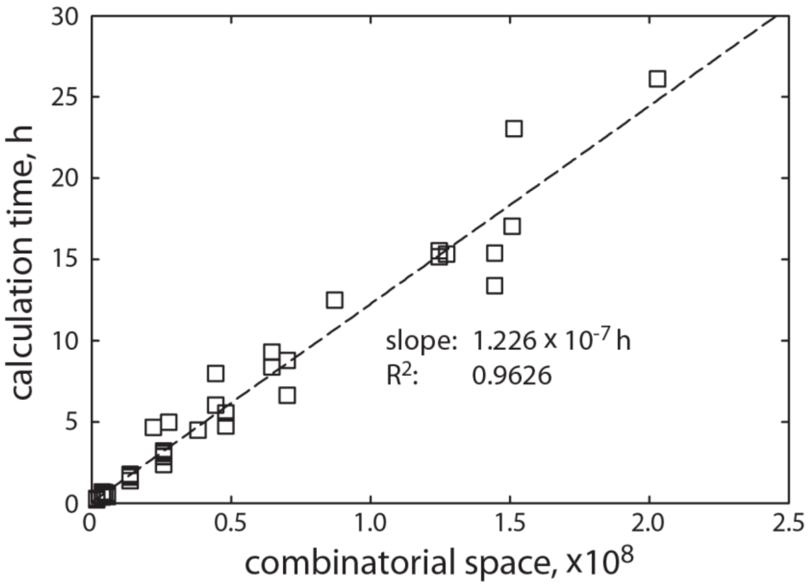

Supplementary Figure 1 | Performance analysis of RedLibs (version 0.2.3). The required calculation time for the evaluation of all sub-libraries with a given target size are plotted against the combinatorial space. Computation of libraries was performed on a grid using 144 cores. The specified slope of the linear regression line represents the approximate time required to evaluate a single sub-library.

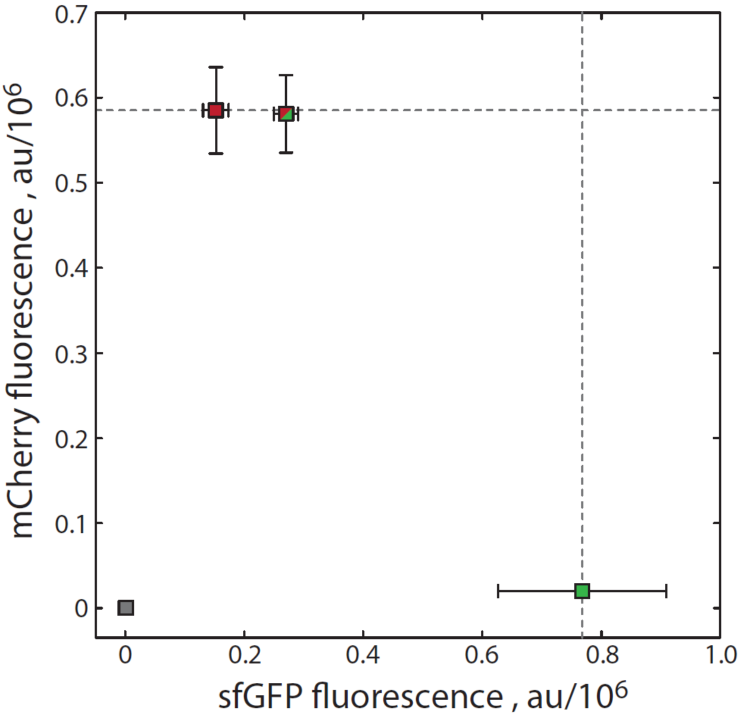

Supplementary Figure 2 | Analysis of mutual influence for combined strong RBS's. The mutant containing the strongest RBS's according to TIR prediction both for sfGFP and mCherry (green-red square) exhibits a strong downshift in the sfGFP signal as compared to a "high green" reference (green square) containing the identical RBS for sfGFP but a weak RBS for mCherry but only a slight downshift in the mCherry channel compared to the "high red" (red square) reference containing the identical RBS for mCherry but a weak RBS for sfGFP. The average fluorescence for the "high green" and "high red" references are indicated by the dashed lines. Data points represent the average cell-specific fluorescence of 16 independent replicate cultures including standard deviation in the green and red channel as horizontal and vertical error bars, respectively. A background control lacking the genes both for sfGFP as well as mCherry was included (grey square).

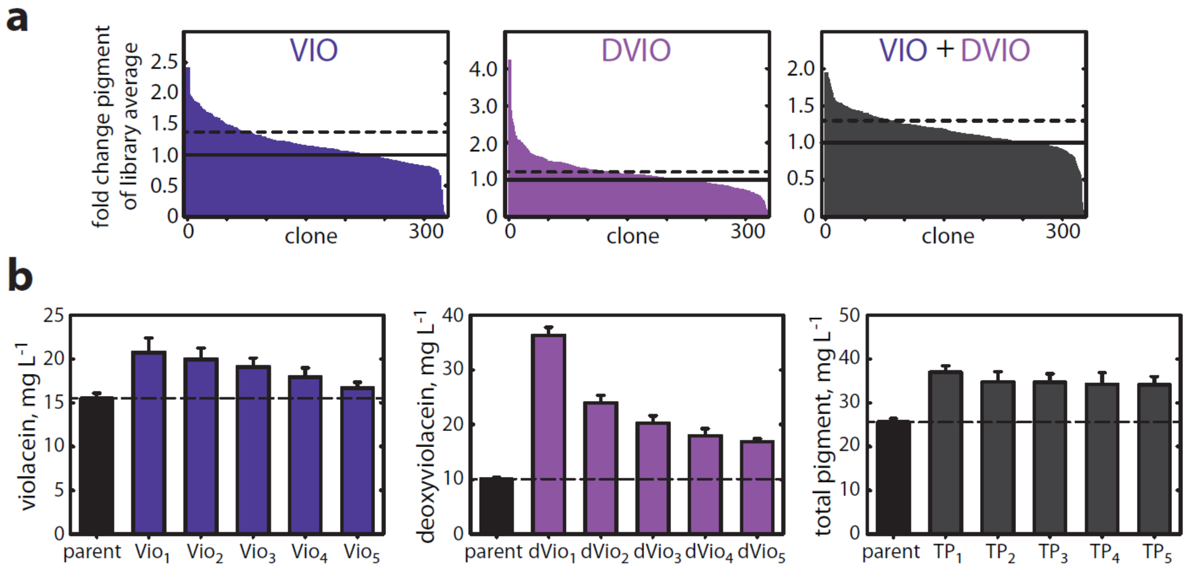

Supplementary Figure 3 | Manipulation of absolute pigment levels of the violacein biosynthesis pathway. (**a**) The rationally reduced RBS library exhibits a large phenotypic variety with respect to absolute pigment levels. Within the limited screening effort (372 clones) mutants with higher levels for violacein, deoxyviolacein and crude pigment as compared to the library average (solid line) as well as the parent clone (dashed line) were identified. (**b**) The five best mutants for violacein, deoxyviolacein and crude pigment production respectively were characterized by quantitative pigment extraction. Data represents the average of four independent replicate cultures with standard deviation.

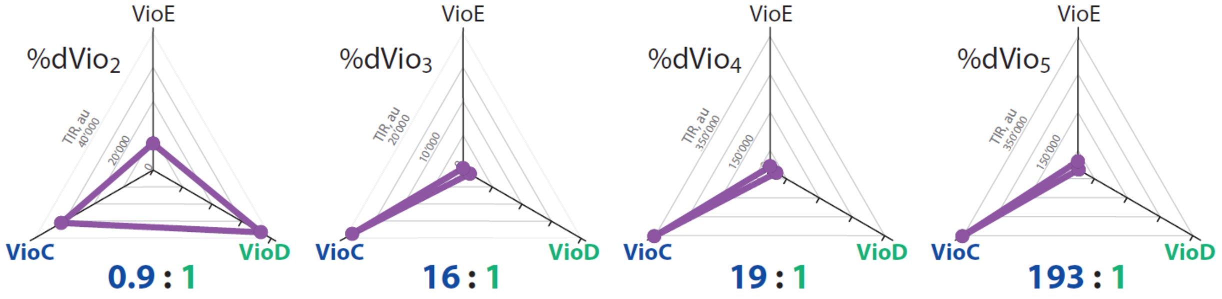

Supplementary Figure 4 | Operon prediction for clones with high selectivity for deoxyviolacein. The TIR's for clones with the highest deoxyviolacein selectivity (clones %dVio$_{2-5}$; prediction value according to[1]) are given. Please note that clone %dVio$_1$ was omitted due to an in-frame stop codon mutation in *vioD* and can therefore be considered a knock-out mutant for this gene.

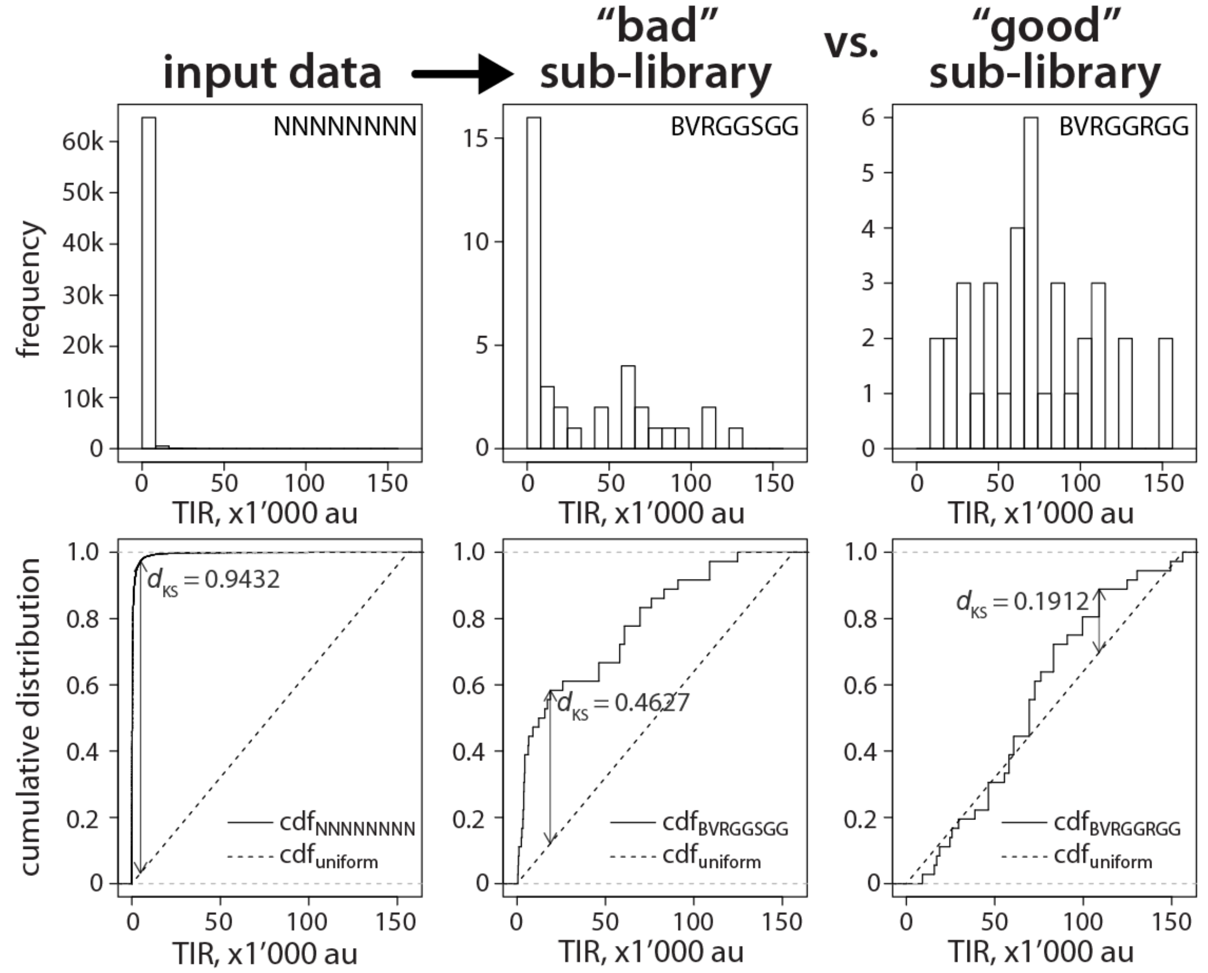

Supplementary Figure 5 | Illustration of the library evaluation performed by RedLibs. The distribution of TIR values assigned to the input data set (NNNNNNNN) of 65'536 sequences predicted[2] for mCherry (see also Results section, Fig. 2) as well as for two reduced sub-libraries with a combinatorial size of 36 (BVRGGSGG and BVRGGRGG) are depicted in the form of histograms (top) and their cumulative distribution functions (bottom). The Kolmogorov-Smirnov distance (d_{KS}) between the library cdf (solid line) and the cdf of a uniform target distribution (dashed line) is highlighted (double arrow) and represents the criterion for library quality: high values of d_{KS} (as for NNNNNNNN or BVRGGSGG) represent unsatisfactory library distributions whereas low d_{KS} values (as for BVRGGRGG) indicate a good resemblance between actual and target distribution. The calculation of cdf's and d_{KS} is described in the Methods section.

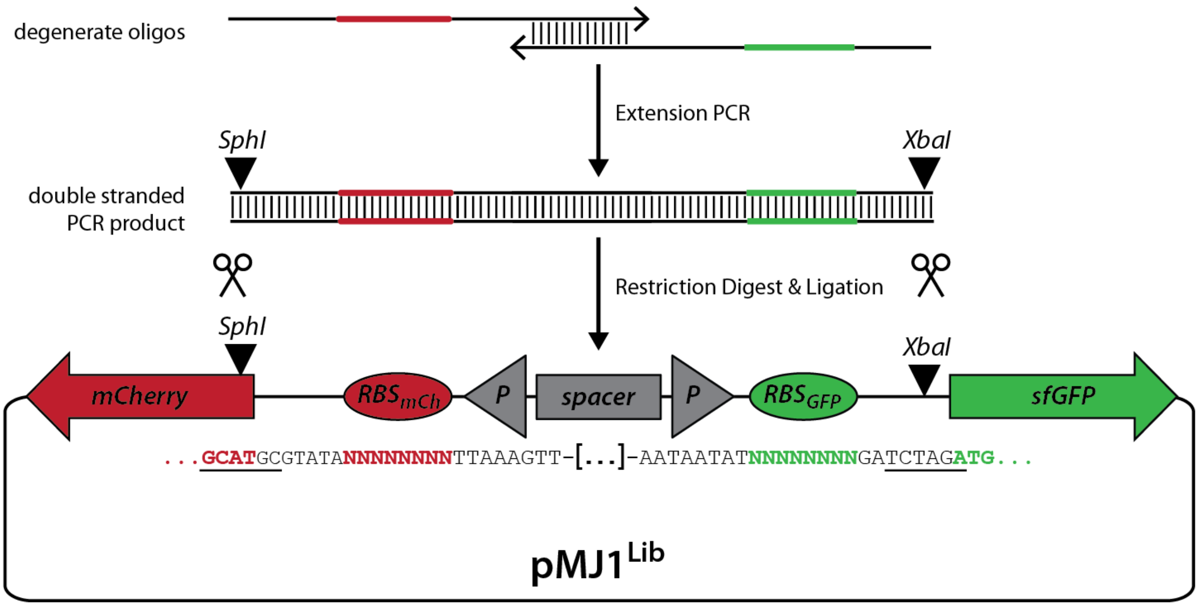

Supplementary Figure 6 | Schematic depiction of the cloning procedure for the XFP RBS libraries. In order to introduce the respective degeneracy into the RBS regions of mCherry and sfGFP two overlapping degenerate oligonucleotides are fused together by extension PCR[3] and the resulting double stranded PCR product is inserted into the target vector pMJ1^{Lib} by conventional restriction digest (*Sph*I & *Xba*I) and ligation procedures. The process is exemplified for oligonucleotides 1+2 resulting in an N_8xN_8 library and was carried out accordingly for oligonucleotides 3+4 (N_6xN_6 library), 5+6 (4x4 library), 7+8 (12x12 library), and 9+10 (24x24 library).

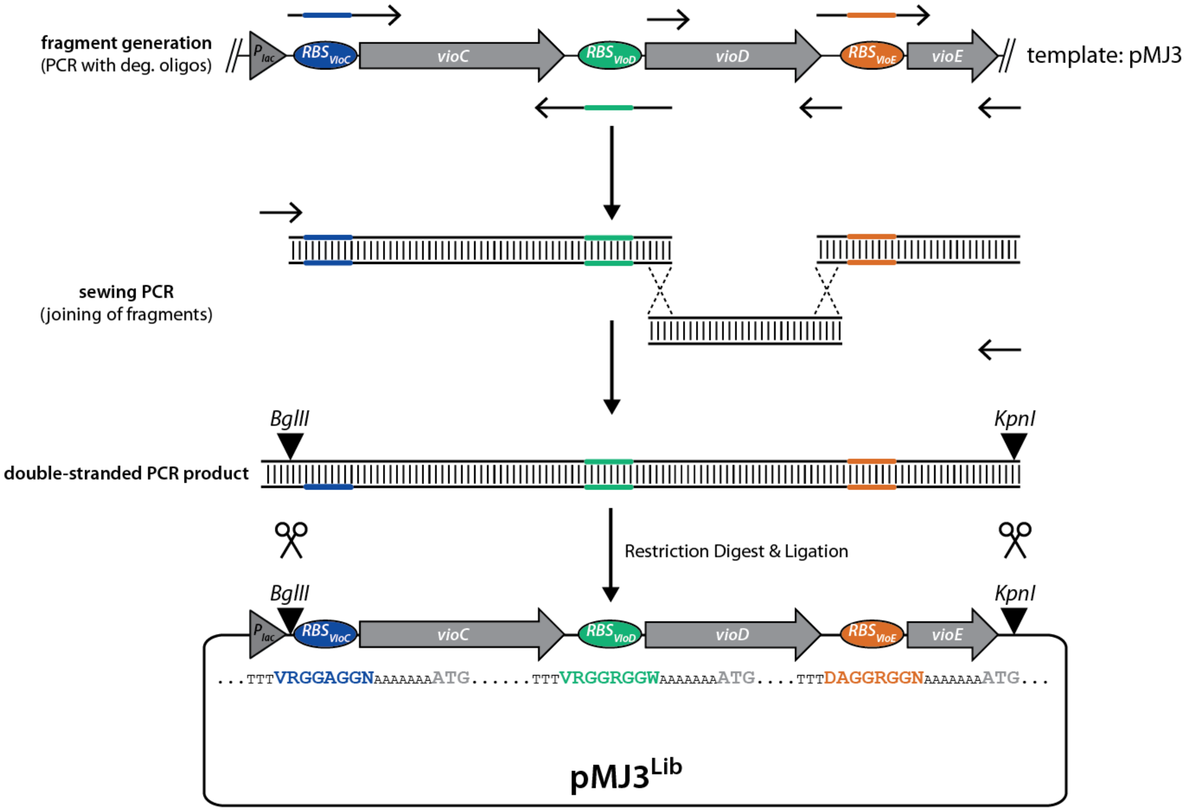

Supplementary Figure 7 | Schematic cloning procedure for the first violacein RBS library. In order to introduce the respective degeneracy into the RBS regions of *vioC*, *vioD* and *vioE* initially three fragments are created by PCR using oligonucleotide pairs 19+20, 21+22, and 23+24 and pMJ3 as template. Subsequently these fragments are joined in a sewing PCR making use of the homologous end regions[3]. The resulting full-length double stranded PCR product is then inserted into the target vector pMJ3^{Lib} by conventional restriction digest (*Bgl*II & *Kpn*I) and ligation procedures. A similar procedure was carried out in order to produce the second violacein RBS library but using oligonucleotide pairs 25+26, 21+22, and 27+24 to generate the three PCR fragments in the first step.

Supplementary Table 1 | Sequences specified for prediction by the RBS Library Calculator. To retrieve input data sets for the respective genes the degenerate RBS region containing eight consecutive N's (underlined) was specified in the RBS library calculator[2]. Additionally 20 bp upstream of the RBS (pre-sequence) as well as the first 50 bp of the coding sequence (start codon highlighted in bold) were used. The 16S-rRNA sequence was chosen for *E. coli* strain DH10B.

Gene	Pre-sequence (20 bp)	Degenerate RBS	Coding Sequence (50 bp)
sfGFP	GATAAATGCTTCAATAATAT	NNNNNNNNGATCTAG	**ATG**AGCAAAGGAGAAGAACTTTTCACTGGAGTTGTCCCAATTCTTGTTGA
mCherry	ATAATTTTGTTTAACTTTAA	NNNNNNNNTATACGC	**ATG**CATAGCAAGGGCGAAGAAGATAACATGGCCATCATCAAGGAGTTCAT
vioC	CTCGGTATCATGAGATCTTT	NNNNNNNNAAAAAAA	**ATG**AAACGTGCGATTATCGTTGGTGGCGGCCTGGCGGGTGGCCTGACCGC
vioD	CGCGTGAATTGATGATCTTT	NNNNNNNNAAAAAAA	**ATG**AAGATTCTGGTCATTGGTGCTGGTCCAGCTGGTCTGGTTTTCGCATC
vioE	TTGCAGCGCTGATGATCATT	NNNNNNNNAAAAAAA	**ATG**GAGAACCGTGAGCCACCACTGTTGCCAGCCCGTTGGAGCAGCGCCTA

Supplementary Table 2 | DNA Sequences obtained from commercial gene synthesis. All synthetic genes used in this study were obtained from Life Technologies (Regensburg, Germany). The sequences are listed with start codons of genes (bold) and important restrictions sites (underlined). The open reading frame for sfGFP is given in reverse complement orientation.

Name/Description	Sequence
cassette for the expression of sfGFP and mCherry	AAGCTTTTATTAAGGCCTTTTGTAGAGCTCATCCATGCCATGTGTAATCCCAGCAGCAGTTACAAACTCAAGAA<br>GGACCATGTGGTCACGCTTTTCGTTGGGATCTTTCGAAAGGACAGATTGTGTCGACAGGTAATGGTTGTCTGGT<br>AAAAGGACAGGGCCATCGCCAATTGGAGTATTTTGTTGATAATGGTCTGCTAGTTGAACGGAACCATCTTCAAC<br>GTTGTGGCGAATTTTGAAGTTAGCTTTGATTCCATTCTTTTGTTTGTCTGCCGTGATGTATACATTGTGTGAGT<br>TAAAGTTGTACTCGAGTTTGTGTCCAAGAATGTTTCCATCTTCTTTAAAATCAATACCCTTTAACTCGATACGA<br>TTAACAAGGGTATCACCTTCAAACTTGACTTCAGCACGCGTCTTGTAGGTCCCGTCATCTTTGAAAGATATAGT<br>GCGTTCCTGTACATAACCTTCGGGCATGGCACTCTTGAAAAAGTCATGCCGTTTCATGTGATCCGGATAACGGG<br>AAAAGCATTGAACACCATAGGTCAGAGTAGTGACAAGTGTTGGCCACGGAACAGGTAGTTTTCCAGTAGTGCAA<br>ATAAATTTAAGGGTGAGTTTTCCGTTTGTAGCATCACCTTCACCCTCTCCACGGACAGAAAATTTGTGCCCATT<br>AACATCACCATCTAATTCAACAAGAATTGGGACAACTCCAGTGAAAAGTTCTTCTCCTTTGCT**CAT**CTAGATCC<br>TTTTTCAATATTATTGAAGCATTTATCGCTAGCACAATACCTAGGACTGAGCTAGCCGTCAAGGATCCAGGTTC<br>TGTTAAGTAACTGAACCCAATGTCGTTAGTGACGCTTACCTCTTAAGAGGTCACTGACCTAACAAGATCTTTGA<br>CGGCTAGCTCAGTCCTAGGTATTGTGCTAGCATAATTTTGTTTAACTTTAAGAAGGAGATATACGC**ATG**CATAG<br>CAAGGGCGAAGAAGATAACATGGCCATCATCAAGGAGTTCATGCGCTTCAAGGTGCACATGGAGGGCTCCGTGA<br>ACGGCCACGAGTTCGAGATCGAGGGCGAGGGCGAGGGCCGCCCCTACGAGGGCACCCAGACCGCCAAGCTGAAG<br>GTGACCAAGGGTGGCCCCCTGCCCTTCGCCTGGGACATCCTGTCCCCTCAGTTCATGTACGGCTCCAAGGCCTA<br>CGTGAAGCACCCCGCCGACATCCCCGACTACTTGAAGCTGTCCTTCCCCGAGGGCTTCAAGTGGGAGCGCGTGA<br>TGAACTTCGAGGACGGCGGCGTGGTGACCGTGACCCAGGACTCCTCCTTGCAGGACGGCGAGTTCATCTACAAG<br>GTGAAGCTGCGCGGCACCAACTTCCCCTCCGACGGCCCCGTAATGCAGAAGAAGACCATGGGCTGGGAGGCCTC<br>CTCCGAGCGGATGTACCCCGAGGACGGCGCCCTGAAGGGCGAGATCAAGCAGAGGCTGAAGCTGAAGGACGGCG<br>GCCACTACGACGCTGAGGTCAAGACCACCTACAAGGCCAAGAAGCCCGTGCAGCTGCCCGGCGCCTACAACGTC<br>AACATCAAGTTGGACATCACCTCCCACAACGAGGACTACACCATCGTGGAACAGTACGAACGCGCCGAGGGCCG<br>CCACTCCACCGGCGGCATGGACGAGCTGTACAAGAGGCCTTAATAAGAATTC
cassette for the expression of *vioC*, *vioD* and *vioE*	AGATCTTTAAGGAGGTAAAAAAA**ATG**AAACGTGCGATTATCGTTGGTGGCGGCCTGGCGGGTGGCCTGACCGCG<br>ATCTACCTGGCGAAGCGTGGCTACGAAGTGCACGTCGTGGAGAAGCGTGGTGATCCTCTGCGCGATCTGAGCTC<br>TTACGTGGACGTTGTTAGCAGCCGTGCGATCGGCGTGAGCATGACCGTTCGTGGTATCAAGAGCGTTTTGGCTG<br>CGGGCATTCCGCGTGCAGAGCTGGATGCGTGTGGCGAACCGATCGTGGCAATGGCTTTCTCCGTGGGTGGTCAG<br>TATCGCATGCGCGAACTGAAGCCGTTGGAGGATTTCCGTCCGCTGAGCTTGAACCGTGCGGCGTTTCAAAAGCT<br>GCTGAACAAATACGCGAACCTGGCAGGCGTTCGTTACTACTTTGAGCATAAGTGCCTGGATGTTGACCTGGATG<br>GTAAGAGCGTGTTGATTCAGGGCAAAGATGGTCAGCCGCAGCGTCTGCAAGGTGACATGATTATCGGTGCGGAT<br>GGCGCCCACAGCGCCGTCCGTCAGGCGATGCAGAGCGGCCTGCGTCGTTTCGAGTTCCAGCAAACGTTCTTCCG<br>CCATGGCTACAAAACCCTGGTTTTGCCGGACGCGCAAGCACTGGGTTACCGTAAAGACACGCTGTACTTTTTCG<br>GCATGGATTCCGGTGGCCTGTTCGCGGGTCGTGCGGCTACGATCCCAGATGGTAGCGTCAGCATCGCCGTTTGC<br>CTGCCGTACTCGGGTAGCCCTTCCCTGACGACCACCGACGAACCGACGATGCGTGCGTTCTTCGATCGTTACTT<br>CGGTGGCCTGCCGCGTGACGCGCGTGACGAAATGCTGCGTCAGTTTCTGGCGAAGCCGAGCAACGACCTGATTA<br>ACGTGCGCTCTAGCACCTTTCACTATAAGGGTAATGTGCTGTTGCTGGGTGATGCTGCGCATGCGACTGCGCCG<br>TTCCTGGGTCAGGGTATGAACATGGCGCTGGAGGACGCCCGCACGTTTGTCGAGCTGCTGGACCGCCACCAGGG<br>CGACCAAGACAAAGCCTTTCCGGAGTTCACGGAGCTGCGCAAAGTCCAGGCAGACGCAATGCAAGACATGGCTC<br>GCGCCAACTATGACGTTTTGAGCTGCTCGAACCCGATCTTTTTCATGCGTGCGCGTTACACGCGTTACATGCAT<br>TCCAAGTTTCCGGGCCTGTATCCGCCGGATATGGCCGAGAAACTGTACTTTACGAGCGAGCCGTACGATCGTCT<br>GCAACAAATCCAGCGTAAACAGAATGTTTGGTACAAGATTGGTCGCGTGAATTGATGATCTTTAAGGAGGTAAA<br>AAAA**ATG**AAGATTCTGGTCATTGGTGCTGGTCCAGCTGGTCTGGTTTTCGCATCCCAACTGAAGCAGGCACGCC<br>CTTTGTGGGCCATTGACATCGTGGAGAAGAATGACGAGCAAGAAGTGCTGGGCTGGGGTGTCGTGCTGCCTGGC<br>CGTCCGGGTCAGCACCCGGCGAACCCGCTGTCCTATCTGGATGCACCGGAGCGTCTGAATCCGCAATTTCTGGA

	GGACTTCAAACTGGTGCATCATAATGAGCCGTCCTTGATGTCCACGGGCGTTTTGTTGTGCGGCGTGGAGCGTC<br>GCGGTCTGGTTCACGCGCTGCGCGATAAGTGCCGCAGCCAAGGCATTGCTATTCGTTTCGAAAGCCCGTTGCTG<br>GAACACGGTGAGCTGCCGCTGGCGGACTATGATCTGGTGGTCCTGGCTAATGGTGTTAATCACAAAACCGCGCA<br>TTTCACCGAGGCTCTGGTCCCGCAGGTGGACTACGGCCGCAATAAGTACATTTGGTATGGCACTAGCCAGCTGT<br>TCGATCAGATGAATCTGGTTTTTCGTACCCATGGTAAAGATATCTTTATCGCGCATGCCTATAAGTATAGCGAT<br>ACCATGAGCACGTTCATTGTCGAATGTAGCGAAGAGACTTACGCACGCGCACGCCTGGGCGAAATGTCCGAAGA<br>GGCGAGCGCAGAATACGTTGCTAAGGTGTTCCAGGCCGAGCTGGGTGGTCACGGCCTGGTGAGCCAGCCGGGTC<br>TGGGTTGGCGTAACTTCATGACGTTGTCTCATGACCGTTGTCATGATGGTAAGTTGGTTCTGCTGGGTGACGCG<br>CTGCAAAGCGGTCACTTTAGCATCGGCCACGGCACCACGATGGCCGTGGTGGTGGCGCAGCTGCTGGTTAAAGC<br>GCTGTGTACCGAAGATGGTGTGCCTGCCGCGCTGAAACGTTTCGAAGAGCGTGCCCTGCCGCTGGTGCAGTTGT<br>TCCGTGGCCACGCAGACAACAGCCGCGTTTGGTTCGAAACCGTCGAAGAGCGCATGCACCTGTCCTCGGCGGAA<br>TTTGTGCAAAGCTTCGACGCACGCCGCAAAAGCCTGCCGCCGATGCCGGAAGCACTGGCGCAGAATCTGCGTTA<br>TGCTTTGCAGCGCTGATGATCATTAAGGAGGTAAAAAAA**ATG**GAGAACCGTGAGCCACCACTGTTGCCAGCCCG<br>TTGGAGCAGCGCCTATGTCTCTTATTGGAGCCCGATGCTGCCGGATGACCAGCTGACCAGCGGCTATTGCTGGT<br>TCGACTATGAACGTGACATCTGTCGTATTGACGGCCTGTTCAATCCGTGGAGCGAGCGTGATACTGGTTATCGC<br>CTGTGGATGTCGGAGGTTGGTAATGCGGCCAGCGGCCGTACCTGGAAACAAAAAGTCGCCTATGGTCGTGAGCG<br>TACCGCCCTGGGTGAACAGCTGTGTGAGCGTCCGCTGGATGATGAGACTGGCCCTTTTGCCGAATTGTTCCTGC<br>CACGCGATGTCCTGCGCCGTCTGGGTGCCCGTCACATTGGCCGTCGCGTGGTTCTGGGTCGCGAAGCGGACGGT<br>TGGCGTTACCAGCGCCCAGGTAAAGGTCCGAGCACCCTGTACCTGGATGCGGCGAGCGGCACTCCACTGCGCAT<br>GGTCACCGGCGATGAAGCGTCGCGTGCAAGCCTGCGTGATTTTCCGAATGTGAGCGAGGCGGAGATCCCGGACG<br>CGGTTTTCGCGGCCAAGCGCTAATAAGGGTACC

Supplementary Table 3 | Plasmids used in this study.

Name	Description	Reference
pCK01	pSC101 ori, Cm^R. The P_{lac} promoter including the LacI operator region from this plasmid was inserted into pMJ2 and pMJ3.	Fernandez et al. (1995)[4]
BBa_J72214-BBa_J72090	P15A ori, Cm^R, Addgene plasmid #40782. Contains *vioABCDE* operon, was used as template for generation of pMJ2 and pMJ3.	Kittleson et al. (2012)[5]
pMJ1	SEVA architecture[6], p15A ori, Kan^R. Contains the genes of sfGFP and mCherry in opposing directions under the control of a constitutive promoter (BBa_J23118, BioBricks parts registry).	This study
pMJ2	SEVA architecture[6], pBR322 ori, Amp^R. Contains the genes *vioA* and *vioB* under the control of a P_{lac} promoter (from pCK01) as well as *lacI* including its own constitutive promoter as found in the pET vectors series (Novagen).	This study
pMJ3	SEVA architecture[6], p15A ori, Kan^R. Contains the genes *vioC*, *vioD* and *vioE* under the control of a P_{lac} promoter (from pCK01).	This study

Supplementary Table 4 | Oligonucleotides used in this study. Oligonucleotides 1-10 were purchased from Integrated DNA Technologies (Coralville, IA, USA). All other oligos were obtained from Sigma Aldrich (Buchs, Switzerland). Oligos containing degenerate base (shown in bold) were obtained in a PAGE purified form.

No.	Sequence (5' to 3')
1	CTTCTCCTTTGCTCATCTAGATC**NNNNNNNN**ATATTATTGAAGCATTTATCGCTAGCACAATACCTAGGACTGAGCTAGCCGTCAAGGATCC AGGTTCTGTTAAGTAACTGAACCCAATGTCGTTAGTGACGCTTACC
2	TCGCCCTTGCTATGCATGCGTATA**NNNNNNNN**TTAAAGTTAAACAAAATTATGCTAGCACAATACCTAGGACTGAGCTAGCCGTCAAAGATC TTGTTAGGTCAGTGACCTCTTAAGAGGTAAGCGTCACTAACGACATTGG
3	CTTCTCCTTTGCTCATCTAGATCC**NNNNNN**AATATTATTGAAGCATTTATCGCTAGCACAATACCTAGGACTGAGCTAGCCGTCAAGGATCC AGGTTCTGTTAAGTAACTGAACCCAATGTCGTTAGTGACGCTTACC
4	TCGCCCTTGCTATGCATGCGTATAT**NNNNNN**CTTAAAGTTAAACAAAATTATGCTAGCACAATACCTAGGACTGAGCTAGCCGTCAAAGATC TTGTTAGGTCAGTGACCTCTTAAGAGGTAAGCGTCACTAACGACATTGG
5	CTTCTCCTTTGCTCATCTAGATCACC**Y**CCT**W**ATATTATTGAAGCATTTATCGCTAGCACAATACCTAGGACTGAGCTAGCCGTCAAGGATCC AGGTTCTGTTAAGTAACTGAACCCAATGTCGTTAGTGACGCTTACC
6	TCGCCCTTGCTATGCATGCGTATA**Y**CTCCTC**M**TTAAAGTTAAACAAAATTATGCTAGCACAATACCTAGGACTGAGCTAGCCGTCAAAGATC TTGTTAGGTCAGTGACCTCTTAAGAGGTAAGCGTCACTAACGACATTGG
7	CTTCTCCTTTGCTCATCTAGATCACC**Y**CC**YH**ATATTATTGAAGCATTTATCGCTAGCACAATACCTAGGACTGAGCTAGCCGTCAAGGATCC AGGTTCTGTTAAGTAACTGAACCCAATGTCGTTAGTGACGCTTACC
8	TCGCCCTTGCTATGCATGCGTATACCTCC**YBR**TTAAAGTTAAACAAAATTATGCTAGCACAATACCTAGGACTGAGCTAGCCGTCAAAGATC TTGTTAGGTCAGTGACCTCTTAAGAGGTAAGCGTCACTAACGACATTGG

9	CTTCTCCTTTGCTCATCTAGATC**M**CC**Y**CC**HY**ATATTATTGAAGCATTTATCGCTAGCACAATACCTAGGACTGAGCTAGCCGTCAAGGATCC AGGTTCTGTTAAGTAACTGAACCCAATGTCGTTAGTGACGCTTACC
10	TCGCCCTTGCTATGCATGCGTATACC**Y**CC**YBR**TTAAAGTTAAACAAAATTATGCTAGCACAATACCTAGGACTGAGCTAGCCGTCAAAGATC TTGTTAGGTCAGTGACCTCTTAAGAGGTAAGCGTCACTAACGACATTGG
11	GAAAAGTTCTTCTCCTTTGCTCATC
12	GCCCTTGCTATGCATGCG
13	TCATGAGATCTTTAAGGAGG
14	TAGGATCCTCATTAGGCCTCGCGGC
15	TAAAGCTTGCAATTAATGTGAGTTAGCTC
16	CCTCCTTAAAGATCTCATGATACCGAGCTCGAATTCG
17	TAACTAGTCACTGCCCGCTTTCCAGTC
18	TAAAGCTTGACACCATCGAATGGCGCAAAAC
19	GAGCTCGGTATCATGAGATCTTT**VR**GGAGG**N**AAAAAAAAATG
20	CAATGACCAGAATCTTCATTTTTTTTT**W**CC**Y**CC**YB**AAAGATCATC
21	AAAAAAAAATGAAGATTCTGGTCATTG
22	AATGATCATCAGCGCTGC
23	GCAGCGCTGATGATCATT**D**AGG**R**GG**N**AAAAAAAAATG
24	GCGAATTCGAGCTCGGTACCC
25	GAGCTCGGTATCATGAGATCTTTAGGG**RR**G**S**AAAAAAAAATG
26	CAATGACCAGAATCTTCATTTTTTTTTACC**Y**CC**YY**AAAGATCATC
27	GCAGCGCTGATGATCATTGAGGAGGTAAAAAAAAATG

Supplementary Methods

Chemicals and Reagents

If not stated otherwise all chemicals and reagents were obtained from Sigma Aldrich (Buchs, Switzerland). Restriction enzymes were obtained from New England Biolabs (Ipswich, MA, USA).

R-Function for *in silico* picking

```
#in silico picking function:

get.sim.distr <- function( distribution, picks ){
  list.indices    <- 1:length( distribution )
  sample.indices  <- sample( list.indices, picks, replace=TRUE )
  picked_values   <- distribution[ sample.indices ]
  return(picked_values)
}
```

Supplementary References

1. Borujeni, A.E., Channarasappa, A.S. & Salis, H.M. Translation rate is controlled by coupled trade-offs between site accessibility, selective RNA unfolding and sliding at upstream standby sites. *Nucleic Acids Res.* **42**, 2646-2659 (2014).
2. Salis, H.M., Mirsky, E.A. & Voigt, C.A. Automated design of synthetic ribosome binding sites to control protein expression. *Nat. Biotechnol.* **27**, 946-950 (2009).
3. Heckman, K.L. & Pease, L.R. Gene splicing and mutagenesis by PCR-driven overlap extension. *Nat. Protoc.* **2**, 924-932 (2007).
4. Fernandez, S., Delorenzo, V. & Perezmartin, J. Activation of the transcriptional regulator XylR of Pseudomonas putida by release of repression between functional domains. *Mol. Microbiol.* **16**, 205-213 (1995).
5. Kittleson, J.T., DeLoache, W., Cheng, H.Y. & Anderson, J.C. Scalable plasmid transfer using engineered P1-based phagemids. *ACS Syn. Biol.* **1**, 583-589 (2012).
6. Silva-Rocha, R. et al. The Standard European Vector Architecture (SEVA): a coherent platform for the analysis and deployment of complex prokaryotic phenotypes. *Nucleic Acids Res.* **41**, D666-D675 (2013).
