# Supplementary figures and images for: Rationally reduced libraries for combinatorial pathway optimization minimizing experimental effort
Source: Nat Commun. 2016 Mar 31;7:11163. doi: 10.1038/ncomms11163 (PMC4821882; doi:10.1038/ncomms11163)

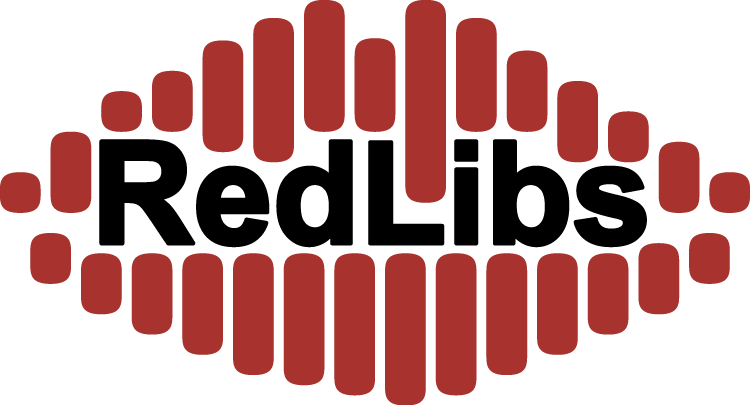

Supplement: Supplementary Software — Sequence-numerical value pair lists of fully degenerate sequences are used as input to calculate smart libraries with a desired distribution encoded by a reduced degenerate sequence. Source code files (C++) for the RedLibs algorithm v1.0.0 including file description are provided. A frequently updated version can be found under the link provided in the Methods section [file ncomms11163-s2.zip › Logo/RedLibs_logo.png]
